# Downregulation of the glucose transporter GLUT 1 in the cerebral microvasculature contributes to postoperative neurocognitive disorders in aged mice

**DOI:** 10.1186/s12974-023-02905-8

**Published:** 2023-10-19

**Authors:** Ying Chen, Jin Joo, John Man-Tak Chu, Raymond Chuen-Chung Chang, Gordon Tin-Chun Wong

**Affiliations:** 1 Department of Anaesthesiology, LKS Faculty of Medicine, Queen Mary Hospital, The University of Hong Kong, Room K424, 4Th Floor, K Block, 102 Pokfulam Road, Pokfulam, Hong Kong SAR China; 2 Laboratory of Neurodegenerative Diseases, School of Biomedical Sciences, LKS Faculty of Medicine, L4-49, Laboratory Block, Faculty of Medicine Building, The University of Hong Kong, 21 Sassoon Road, Pokfulam, Hong Kong SAR China; 3 Department of Anesthesiology, First Affiliated Hospital, Sun Yat-Sen University, Guangzhou, China; 4 Department of Anaesthesia and Pain Medicine, College of Medicine, Seoul St. Mary’s Hospital, The Catholic University of Korea, 222 Banpodaero, Seocho-Gu, Seoul, 06591 Korea; 5 State Key Laboratory of Brain and Cognitive Sciences, The University of Hong Kong, Hong Kong SAR, China

**Keywords:** Postoperative neurocognitive disorders, GLUT1, Glucose metabolism, Blood–brain barrier, Aging

## Abstract

**Introduction:**

Glucose transporter 1 (GLUT1) is essential for glucose transport into the brain and is predominantly expressed in the cerebral microvasculature. Downregulation of GLUT1 precedes the development of cognitive impairment in neurodegenerative conditions. Surgical trauma induces blood–brain barrier (BBB) disruption, neuroinflammation, neuronal mitochondria dysfunction, and acute cognitive impairment. We hypothesized that surgery reduces the expression of GLUT1 in the BBB that in turn disrupts its integrity and contributes to metabolic dysregulation in the brain that culminates in postoperative cognitive impairment.

**Methodology:**

Using an abdominal surgery model in aged WT mice, we assessed the perioperative changes in cognitive performance, tight junction proteins expression, GLUT1 expression, and the associated metabolic effects in the hippocampus. Thereafter, we evaluated the effects of these parameters in aged mice with conditional overexpression of GLUT1, and then again in aged mice with conditional overexpression of GLUT1 with or without prior exposure to the GLUT1 inhibitor ST-31.

**Results:**

We showed a significant decline in cognitive performance, along with GLUT1 reduction and diminished glucose metabolism, especially in the ATP level in the postoperative mice compared with controls. Overexpression of GLUT1 expression alleviated postoperative cognitive decline and improved metabolic profiles, especially in adenosine, but did not directly restore ATP generation to control levels. GLUT1 inhibition ameliorated the postoperative beneficial effects of GLUT1 overexpression.

**Conclusions:**

Surgery-induced GLUT1 reduction significantly contributes to postoperative cognitive deficits in aged mice by affecting glucose metabolism in the brain. It indicates the potential of targeting GLUT1 to ameliorate perioperative neurocognitive disorders.

**Supplementary Information:**

The online version contains supplementary material available at 10.1186/s12974-023-02905-8.

## Introduction

Perioperative neurocognitive disorders (PNDs) commonly develop after surgery and are characterized by a spectrum of cognitive dysfunction, including memory, concentration, learning and comprehension impairment [1, 2]. Advanced age is one of the major risk factors for PNDs, as elderly patients usually have more comorbidities and are more likely to have some preexisting cognitive impairment [2, 3]. Globally there is an increasing number of patients undergoing surgery, with more than one-third of those performed being on patients over 65 years of age [4]. However, effective preventive or therapeutic options are currently unavailable [5] and thus, PNDs, especially in the aging population is a great but underappreciated public health concern.

The blood–brain barrier (BBB) is a tightly sealed endothelium layer within the brain’s blood vessels that ensures a functional and molecular separation of the brain from the periphery and protect neurons against pathogens and toxins [6, 7]. Its dysfunction has been characterized in several neurodegenerative conditions and age-dependent cognitive decline [6–8]. Senescence in the cerebral vasculature can result in the breakdown of the BBB and increases the risk for developing various neurological conditions [9]. Recent studies suggest that surgery-induced immune responses downregulate tight junctions and disrupts their integrity, thus facilitate infiltration of peripheral immune cells and mediators into the brain and promote neuroinflammation and neuronal damage [10–12]. Our previous studies have demonstrated that peripheral and central immune responses contribute to cognitive impairment in both adult and aged mice in postoperative period [13, 14]. Further evidence has also shown the BBB disruption in aged individuals receiving different types of surgery or even anesthesia only [15–17].

Glucose is a primary energy substrate for neurons and the glucose transporter GLUT1 is of special importance for glucose transport into the brain [18, 19]. Normal GLUT1 expression is important for neuronal function and neurodevelopment. In the central nervous system (CNS), GLUT1 is exclusively expressed by cells in the BBB, but not by neurons [20]. GLUT1 has two isoforms: one 55 kDa isoform in endothelial cells, the other one a 45 kDa isoform present at the end-feet of adjacent astrocytes [21]. The level of endothelial GLUT1 in the BBB determines brain glucose uptake and the activity of the transporter is independent on insulin signaling [22]. Consequently, impaired GLUT1 expression or activity correlates with insufficient glucose metabolism in the brain [23]. Homozygous GLUT1 knockout mice die in utero with visible developmental abnormalities, while heterozygous GLUT1 knockout mice have reduced GLUT1 protein levels and manifest with decreased glucose uptake, lower glucose concentration in the cerebrospinal fluid. In adulthood these mice also develop neurological disorders such as epilepsy, movement disorders and cognitive impairment and introduction of a ketogenic diet at very early stage only partially alleviates these functional disorders [21]. Furthermore, downregulation of GLUT1 is associated with cognitive impairment in neurodegenerative diseases, especially in Alzheimer’s Disease (AD) [24], with subtle GLUT1 deficiencies being present much earlier than the onset of symptoms [23]. A recent study has shown that GLUT1 deficiency impaired cerebral blood flow and glucose uptake, resulting in the breakdown of the BBB and accelerated Aβ accumulation and cognitive impairment in AD mice [25], suggesting a critical role for GLUT1 in maintaining blood–brain barrier function and integrity.

Glucose metabolism is essential for maintaining neuronal function by generating ATP, an essential energy substrate for the biosynthesis of amino acids and neurotransmitters [26, 27]. Dysregulated glucose metabolism is a key step in the development of neurodegenerative diseases and dementia and precedes the onset of cognitive symptoms [28, 29]. Surgery has been shown to induce mitochondrial dysfunction and cognitive impairment in aged mice, which may in part reflect metabolic dysregulation in the brain and this may contribute to the development of PNDs [30].

While BBB disruption, GLUT1 deficiency, glucose dysregulation and mitochondria dysfunction are well demonstrated in various conditions with cognitive impairment, their interrelationships remained relatively unexplored especially in the context of PNDs. In this study we hypothesized that surgery reduces the expression of GLUT1 in the BBB that in turn disrupts it integrity and contributes to metabolic dysregulation in the hippocampus that culminates in postoperative cognitive impairment. To test this hypothesis, GLUT1 expression in the blood–brain barrier, tight junction levels, and hippocampal glucose metabolism after surgery were initially evaluated in an aged mouse model of PNDs. This was followed by exploring the effects of overexpressing endothelial GLUT1 in the brains of preoperative animals on postoperative cognitive function, hippocampal glucose metabolism and blood–brain barrier integrity.

## Materials and methods

### Ethics declarations

Animal protocols were approved by the Department of Health, Hong Kong and Committee on the Use of Live Animals in Teaching and Research, The University of Hong Kong. Male C57BL/6N wild type (WT) mice aged between 18 and 20 months were obtained from the Center of Comparative Medicine Research of the University of Hong Kong. The handling of animals and all experimental procedures were conducted in accordance with National Institutes of Health guide for the care and use of Laboratory animals and Animals (Control of Experiments) Ordinance, Hong Kong, China.

The mice were used in three set of experiments. The first set evaluated the changes in cognitive function, blood–brain barrier integrity and hippocampal metabolism by assigning the mice into three groups: no anesthesia or surgery as control (CON), exposure to sevoflurane anesthesia only (SEVO) and laparotomy under sevoflurane anesthesia (LAP). For the second set of experiments, the animals were assigned into 4 groups to evaluate the effects of enhanced endothelial GLUT1 expression on postoperative cognitive function: sevoflurane anesthesia with control AAV9 vector (SEV + AAV9-ICAM2-CON), sevoflurane anesthesia with conditional GLUT1 overexpression vector (SEV + AAV9-ICAM2-GLUT1), laparotomy with control AAV9 vector (LAP + AAV9-ICAM2-CON) and laparotomy with conditional GLUT1 overexpression vector (LAP + AAV9-ICAM2-GLUT1).

In the third set of experiments, animals subjected to enhanced GLUT1 expression and a laparotomy and were further divided into two groups: one treated with GLUT1 specific inhibitor STF-31 (LAP + GLUT1 + STF-31) and the other treated with vehicle (LAP + GLUT1 + vehicle) to further confirm the role of GLUT1 on cognitive functions.

### Laparotomy

A laparotomy was performed under sevoflurane anesthesia, as described previously with minor modifications [14]. General anesthesia was induced and maintained at 3% sevoflurane with 1L/min of oxygen. A 2.5 cm longitudinal incision was made in the midline of the abdomen. Approximately 10 cm of intestine was exteriorized, gently rubbed with fingers for 2 min and replaced into the abdominal cavity after a one-minute’s interval. Absorbable and non-absorbable sutures were used to close the muscle layers and skin, respectively. During the perioperative period, the respiratory rate and the color of the paws were monitored continuously, and the body temperature was maintained by using the heating pad. The duration of the operative procedure was 25 min or less. Buprenorphine was given twice daily for the first 3-day post-operative days for analgesia. The sevoflurane group received 3% sevoflurane for 25 min under the same conditions.

### Construction of a conditional GLUT1 overexpression AAV9 vector

The coding sequence of GLUT1 was cloned downstream of the endothelial-specific promotor ICAM2 in the AAV transfer plasmid (Vigenbio, China) and a green fluorescent protein (GFP) sequence was inserted after the coding sequence to indicate transfection and titer assay by RT-PCR. The control plasmid has a similar structure containing the ICAM2 promoter and GFP sequence but not the GLUT1 coding sequence. The overexpression efficiency of the GLUT1 sequence was verified in HEK293T cultured cells.

The AAV9 viral vector was produced according to triple plasmid protocols. HEK293T cells were transfected with the constructed AAV transfer plasmid containing GLUT1 sequence, pAdDeltaF6 (Addgene, USA) and pAAV2/9n (Addgene, USA) at a ratio of 1:1:1 with the facilitation of Lipofectamine 3000 (Thermo, USA), when the cell confluence reached approximately 70%. At 6 h after post-transfection at 37 °C, the medium was replaced with fresh culture media containing 5% fetal bovine serum. After 96 h of incubation at 37 °C, the cells were harvested and centrifuged at 2000*g*. The viral solution was extracted and purified using the AAVpro purification kit (Takara, Japan, Cat. # 6675). The titers of control AAV9 vector (AAV9-ICAM2-CON) and conditional GLUT1 overexpression AAV9 vector (AAV9-ICAM2-GLUT1) reached to 2–3 × 10^12^ v.g/ml, which were quantified by real-time PCR using the pair of GFP primer: forward 5′-GCA TCG ACT TCA AGG AGG AC-3′ and reverse 5′-GAA CTC CAG CAG GAC CAT GT-3′.

### Stereotaxic injections

These were performed under general anesthesia induced by ketamine (80–100 mg/kg) and xylazine (5–10 mg/kg). The mice were fixed onto the stereotaxic apparatus, and a 2 cm longitudinal incision was made in the middle of the prepared scalp to expose the bregma. Two microliters of AAV9 viral vector (2–3 × 10^12^ v.g/ml) was slowly injected into the lateral ventricle at the speed of 1ul/min, using these coordinates: AP(Bregma) − 0.6 mm; ML: ± 1.3 mm; DV: + 3 mm. Nonabsorbable sutures were applied to close the scalp. The mouse was then transferred to a clean cage for recovery from anesthesia in the postoperative care area. Meloxicam containing drinking water was given for 3 days as post-operative analgesia.

### Intranasal administration of STF-31

From postoperative days 8 to 14, intranasal administration of STF-31 were conducted twice per day for first 3 days and then once per day for the remaining 4 days as previous described [31] with minor modifications. The GLUT1 specific inhibitor STF-31 (1.2 mg/ml, MCE, USA) was dissolved in 10% DMSO, 40% PEG400, 45% saline and 5% Tween-80. The mouse was held by hand while a total of 10ul of the drug or vehicle was ejected using a pipettor into the mouse’s nostril.

### Open field test (OFT)

This is a classical behavior test used to evaluate locomotor activity and the level of anxiety in rodents based on their spontaneous and exploring activities. The protocol was conducted as previously described [32]. After 30-min of pre-habituation in a dimly lit environment, the animals were allowed to freely explore for 10 min in an empty square arena measuring 40 cm × 40 cm × 40 cm and their movements were video recorded. The arena was divided equally into 25 square zones with the middle 9 zones demarcated as the central area. The total exploration time spent in the central area indicates the animal’s level of anxiety and depression and the total exploration distance indicates locomotor activity.

### Forced alternation Y-maze (Y-maze)

This Y-maze test is used for determining hippocampus-dependent memory function as previously described [30]. This test was conducted in a symmetrical Y shaped device, containing two dark arms that can deliver electric shocks and one white shock-free arm as the safe arm. During preoperative training, after 10-min habituation in the apparatus, the mouse was placed alternatively in one end of dark arms and forced to enter the white arm by electric shocks delivered at 2 Hz and 40 ± 5 V for 10 s. If the mouse fails to make a correct choice within 10 s, it would be assisted to enter the white arm and stay there for 30 s. Successful training was considered when the mouse makes 9 consecutive correct choices. During postoperative testing, the mouse was tested on 10 occasions. The number of incorrect choices (number of error) and the time taken (latency) to enter the white arm were recorded to evaluate the rodent’s learning and memory function.

### Novel object recognition test (NOR)

This NOR test was conducted as previously described [30]. Twenty-four hours prior to the test, the animal was allowed to habituate for 10 min in an empty square box measuring 40 cm × 40 cm × 40 cm placed in an evenly lit environment. On day 1 for familiarization, two identical objects (A1 + A2) were placed symmetrically in the arena and the animal was allowed to freely explore them for 10 min. On day 2 for discrimination testing, one of object (A) was replaced with a novel object (B), and the mouse was placed back into the same arena and allowed to freely explore the objects (A + B) for 10 min. The exploration on day 2 were recorded and object interactions was defined by the mouse pointing its nose at and/or touching the object. The discrimination index is the ratio of the exploration time with novel object (B) over the sum of the total exploration time with objects (A + B), which is a measure of recognition memory.

### Cerebral micro-vessel extraction

We extracted hippocampal micro-vessels as previously described with minor modifications [33]. The brain tissue was quickly frozen in liquid nitrogen after harvesting, and later thawed on ice and homogenized with a Dounce homogenizer in 3 ml of cold sucrose buffer (0.32 M sucrose, 5 mM HEPES, pH 7.4). The brain homogenate was centrifuged at 1000*g* for 10 min. After discarding the supernatant, the pellet was resuspended with 3 ml of cold sucrose buffer and centrifuged at 1000*g* for 10 min. These steps were repeated twice. Finally, the pellet was resuspended in 1 ml of cold sucrose buffer, transferred to 1.5 ml Eppendorf tube and centrifuged at 350*g* for 10 min. Enrichment of cerebral micro-vessels was further evaluated by Western blot.

### Western Blot

Protein fractions from synaptosomes and cerebral micro-vessels were subjected to 8–12% SDS-PAGE gel electrophoresis and transferred onto PVDF membranes (Bio-rad, USA). After blocking with 10% non-fat milk solution, the membranes were incubated with specific primary antibodies (PSD95, synapsin I and β-tubulin were from Cellsignaling, USA; synaptophysin and GLUT1 were from Millipore, Germany; synaptobrevin, Synaptic system, Germany; β-actin, Sigma, USA) overnight at 4 °C and subsequently incubated with HRP-conjugated secondary antibodies for 2 h at room temperature. The protein bands were visualized by enhanced chemiluminescence reagents and captured by ChemiDocTM Touch Imaging System (Bio-Rad, USA). The intensity of bands was quantified by ImageJ (WB raw blots were provided in Additional file 3).

### Quantitative real-time polymerase chain reaction (PCR)

Total mRNA from the dissected brain tissue was isolated according to the protocol of Trizol using organic solvents including chloroform, isopropanol and ethanol. The RNA pellet was dissolved in RNase-free water, the quality and concentration were examined by Nano Drop 2000 spectrophotometer. Only those mRNA samples with the ratio of OD260/280 being between 1.8 and 2.0 were qualified to be converted into complementary DNA (cDNA). The relative mRNA level of target genes was assessed by real-time PCR with their specific primers: GLUT1 forward: GCGGGAGACGCATAGTTACA, GLUT1 reverse: CAGCCCCGTTAC TCACCTTG; β-actin forward: GTGGATCAGCAAGCAGGAGT; β-actin reverse: ACGCAGCTCAGTAACAGTCC.

### Immunofluorescent staining

Harvested brains were fixed in 4% PFA for 24 h at 4 °C, followed by 20–30% sucrose solution dehydration. The tissue was then embedded in O.C.T and sectioned into 20 μm-thickness coronal slices. The brain sections were incubated in blocking buffer (PBS with 5% BSA and 0.3% Triton™-100) at room temperature for an hour, then incubated with specific primary antibodies (GLUT1, Millipore, Germany; ZO-1 and Claudin 5 were from Thermo Fisher Scientific, USA) overnight at 4 °C. After rinsing with PBS thrice, the sections were incubated with secondary antibodies conjugated with fluorochrome for 2 h at room temperature and mounted in prolong gold mounting medium with DAPI. The Z-stacks of fluorescent signals were captured at 0.3um steps by Zeiss LSM 880 confocal microscopy. The quantification of fluorescent intensity was processed by using ImageJ.

### Golgi staining analyses

The Golgi-Cox system was used for Golgi staining following the protocol of Hito Golgi-Cox OptimStain™ PreKit. The fresh brain sample was rinsed with ddH_2_O, transferred into the 5 times volume of tissue of impregnation solution that was freshly prepared 24 h beforehand and together stored in the dark at room temperature for 2 weeks, with one change of fresh impregnation solution during the first 24 h. The tissue was then transferred to solution 3 from the kit at 4 °C for 12 h in the dark. At that point fresh solution 3 was then introduced and the tissue was stored at 4 °C for another 24–72 h in the dark. After that, the tissue was embedded with O.C.T and sectioned into 150um thickness slices in a cryostat chamber at − 19 °C. The slices were gently placed onto gelatin-coated slides, and air dry at room temperature in the dark overnight. After rinsing with ddH_2_O, slices were incubated with the staining solution mixture for 10 min, followed by rinsing twice with ddH_2_O. These slices were dehydrated using gradient ethanol, cleared in xylene, and mounted in resinous mounting medium. Then slices were imaging under microscopy after drying, followed by Sholl analysis using Image J.

### Targeted quantitation of glycolysis and tricarboxylic acid cycle metabolites

The fresh hippocampal tissues were dissected on ice, washed by ddH_2_O, and were quickly transferred into liquid nitrogen. Before sample preparation, the weight of the brain tissue was measured, and only the tissue greater than 20 mg was used for the following steps. The sample processing and gas chromatography-tandem mass spectrometry (GC–MS/MS) analyses were performed at Proteomics and Metabolomics Core, Centre for PanorOmic Sciences, LKS Faculty of Medicine, University of Hong Kong. The steps of sample processing and data acquisition are as described.

After tissue homogenization, the polar metabolites were extracted by probe sonication in 500ul methanol/water with 200 ng norvaline internal standard/50 mg tissue and 250 μl of 0.1 M HCl. This was followed by centrifugation at 14,000*g* for 20 min at 40 °C. 50% supernatant was dried before derivatization. The dried residue was redissolved and derivatized for 2 h at 37 °C in 40 μl of hydrochloride (30 mg/ml in pyridine), followed by trimethylsilylation for 1 h at 37 °C in 70 μl MSTFA with 1% TMCS. Up to 1 μl sample was injected for GC–MS/MS analysis.

GC–MS/MS chromatogram was acquired in SCAN and MRM mode in an Agilent 7890B GC-Agilent 7010 Triple Quadrapole Mass Spectrometer system (Santa Clara, CA, USA). The sample was separated through an Agilent (Santa Clara, CA, USA) DB-5MS capillary column (30 m × 0.25 mm ID, 0.25 μm film thickness) under constant flow at 1 ml/min. The GC oven program started at 60 °C (with a hold time 1 min) and was increased 10 °C/min to 120 °C, then 3 °C/min to 150 °C, 10 °C/min to 200 °C and finally 30 °C/min to 280 °C (hold 5 min). Inlet temperature and transfer line temperature were set at 250 °C and 280 °C, respectively. Characteristic quantifier and qualifier transitions were monitored in MRM mode during the run. Mass spectra from m/z 50–500 were acquired in SCAN mode.

Data analysis was performed using the Agilent MassHunter Workstation Quantitative Analysis Software. Linear calibration curves for each analyte were generated by plotting peak area ratio of external/internal standard against standard concentration at different concentration levels. Analytes were confirmed by comparing the retention time and ratio of characteristic transitions between the sample and standard.

### Statistical analysis

GraphPad Prism (version7.0) was used for statistical analyses. All data was assessed by Shapiro–Wilk normality test to check for normal distribution and were presented as mean ± SEM where applicable. Normal distribution was assessed by Shapiro–Wilk normality test. For continuous data, statistical significance was evaluated by one-way ANOVA and followed by Tukey’ multiple comparison tests. For grouped data that consists of two variables such as gene manipulation and surgery or repeated measure data such as blood glucose and body weight, two-way ANOVA followed by Bonferroni’s multiple comparisons test was applied. For discrete data such as the number of errors in the behavioral tests, Kruskal–Wallis test followed by Dunn’s multiple comparisons test was used. *P < 0.05, **P < 0.01, ***P < 0.001.

## Results

### Surgery induced cognitive decline in aged mice

**Fig. 1:**
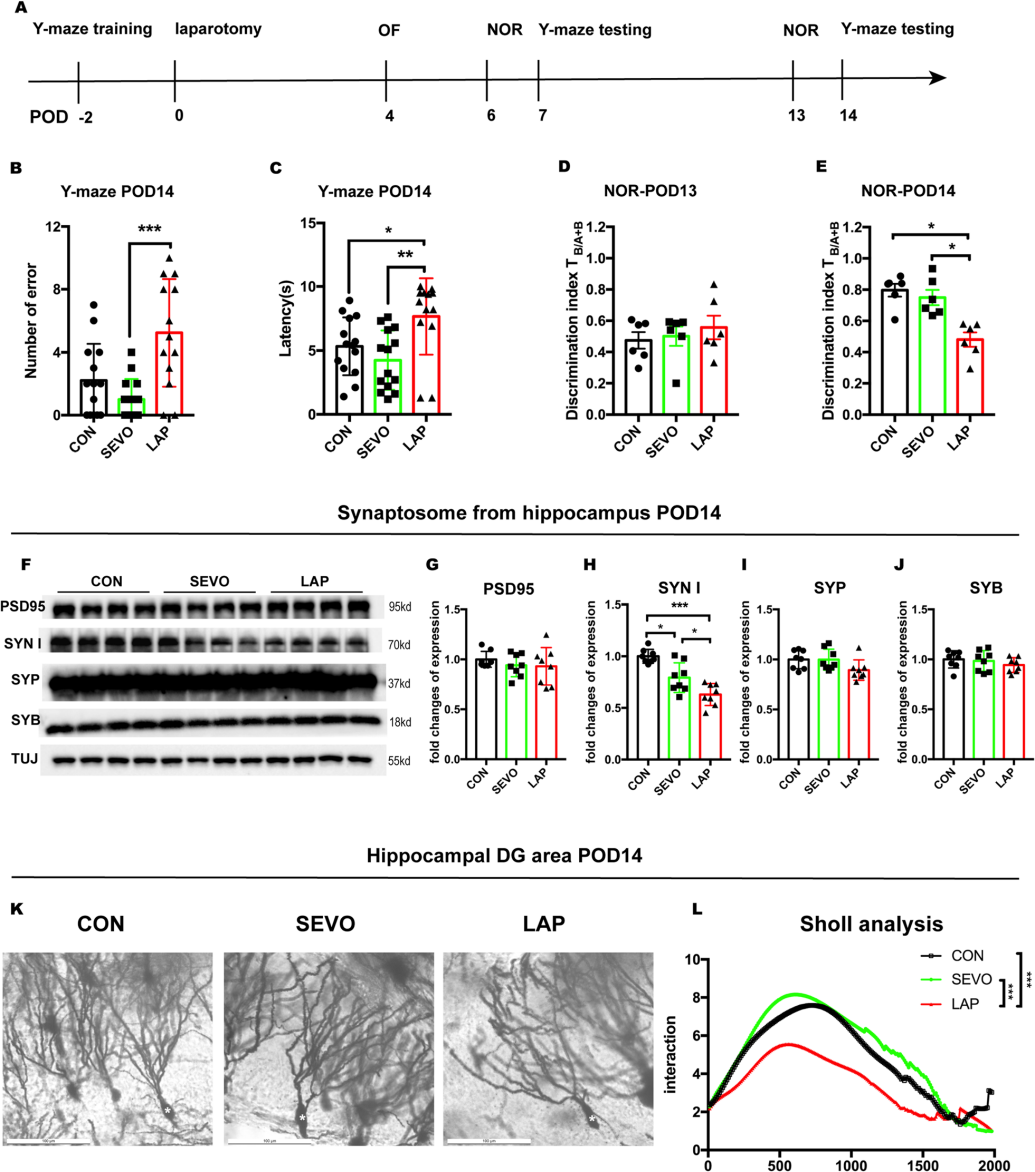
Postoperative changes in cognitive function, synaptic density and neuronal morphology in aged mice. **A** Schema of the experimental design. **B**, **C** Y-maze test on 14, the number of errors (**B**); the latency (**C**). One-way ANOVA with Tukey’s multiple comparison test was applied to the analysis of latency, Kruskal–Wallis test with Dunn’s multiple comparisons test was applied to the number of errors, *n =* 13–14 mice per group. **D**, **E:** NOR test on POD 14, including: discrimination index of two similar objects (A1 and A2) on POD 13 training (**D**); discrimination index of novel object (B) on POD 14 testing (**E**). One-way ANOVA with Tukey’s multiple comparison test with *n =* 6 mice per group. **F**: representative WB images of pre- and post-synaptic markers from hippocampal synaptosomes from POD 14. **G-J**: statistical analysis of pre- and post-synaptic protein levels including: PSD95 (**G**), SYN I (**H**), SYP (**I**), and SYB (**J**), normalized to β-tubulin, One-way ANOVA with Tukey’s multiple comparison test, *n =* 7–8 mice per group. **K:** representative images of Golgi staining in the hippocampus, * representative neurons, scale bar: 100um. **L**: Sholl analysis evaluating neuronal morphology. One-way ANOVA with Tukey’s multiple comparison test, 6 neurons per mice were analysed, *n =* 4 mice per group. Data is presented as mean ± SEM, *P < 0.05, **P < 0.01, ***P < 0.001. POD: post-operative day, Y-maze test = forced alternation Y-maze test, NOR test = novel object recognition test,. PSD95: postsynaptic density protein 95, SYN I: synapsin I, SYP: synaptophysin, SYB: synaptobrevin, TUJ, β-tubulin. CON = control, SEVO = sevoflurane, LAP = laparotomy

Before assessing postoperative cognitive function, we conducted the OFT on POD 4 to determine the presence of any anxiety or locomotor impairment that may interfere with subsequent cognitive testing. No significant differences were found in the central duration time or the total distance travelled between the 3 groups, indicating that neither sevoflurane anesthesia nor laparotomy induced anxiety-like behavior or locomotor impairment (Additional file 1: B, C). We then evaluated learning and memory using the Y-maze and NOR tests. Mice in the LAP group had a longer latency compared to the CON group on POD 7 (Additional file 1: D, E) and had a longer latency and a larger number of errors than the CON and SEVO groups on POD 14 (Fig. 1B, C). These results indicate that postsurgical mice developed problems recognizing the correct (shock free) arm of the Y-maze from POD 7 onwards. Moreover, mice in the LAP group had significantly less interactions with the novel object compared the CON group when tested on POD 7 (Additional file 1: G), and a similar reduction compared to the CON and SEVO groups on POD 14 (Fig. 1K), with no preference of object or location in each advance NOR familiarization sessions (Additional file 1: F; Fig. 1D). The NOR results indicated postsurgical mice had difficulties remembering the familiar object. Results from these behavioral tests suggest that surgery caused cognitive impairment in aged mice at both timepoints while sevoflurane probably had a mild impact on postsurgical mice only at the earlier POD 7 timepoint.

### Surgery decreased synaptic density and altered neuronal morphology in the hippocampus

Appropriate levels of synaptic proteins are important in maintaining normal neuronal and cognitive function. Disrupted synaptic structures have been reported as pathological changes in neurodegenerative diseases. Therefore, we assessed the levels of various synaptic proteins in the hippocampus in postsurgical aged mice. The results showed that pre-synaptic marker SYN I was reduced by sevoflurane, and this reduction was more marked following surgery (Fig. 1F, H), while no significant differences were found in other synaptic markers: PSD95, SYP and SYB (Fig. 1F, G, I, J).

We further investigated and quantified the postsurgical changes in neuronal morphology in the hippocampus using Golgi staining and Sholl analysis (Fig. 1K). The Sholl analysis of the hippocampal DG area showed that the area under the curve (AUC) was smaller in the LAP group when compared to the SEVO and CON groups, both of which had similar AUCs (Fig. 1L). Neuronal complexity such as the total interaction of axons with concentric circles, total axonal length and branch numbers in the LAP group were all decreased compared the SEVO groups, while only the branch numbers were decreased relative to both the CON and SEVO groups (Additional file 1: H–J). These morphological changes of impaired neuronal complexity are consistent with the reduction of synapse density.

### Surgery induced blood–brain barrier impairment in aged mice

**Fig. 2:**
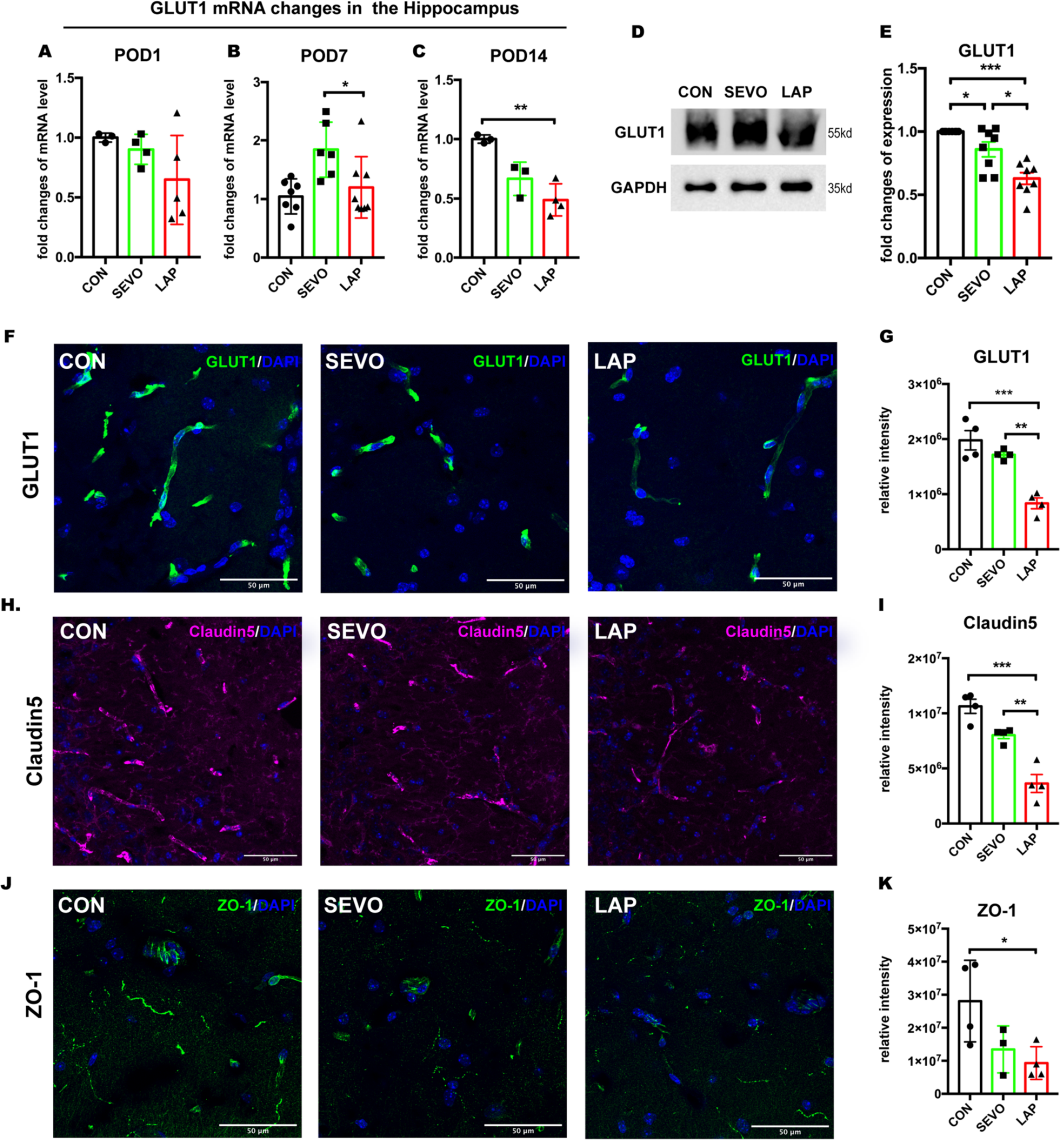
GLUT 1 downregulation and impairment of the blood–brain barrier in postsurgical aged mice. **A**–**C** GLUT1 mRNA expression in the hippocampus on (**A**) POD 1, *n =* 3–5 mice per group; (**B**) POD 7, *n =* 6–7 mice per group and (**C**) POD 14, *n =* 3–4 mice per group; one-way ANOVA with Tukey’s multiple comparison test. **D** Western blot representative images of GLUT1 from cerebral micro-vessel on POD 14. **E** GLUT1 expression from cerebral micro-vessel on POD 14, One-way ANOVA with Tukey’s multiple comparison test with *n =* 8 mice per group. **F** representative confocal images showing GLUT1 staining on POD 14, scale bar: 100um; **G** histogram showing quantification of GLUT1^+^ relative intensity on POD 14. **H** Representative confocal images showing tight junction marker claudin 5 staining on POD 14, scale bar: 50um; **I** Histogram showing quantification of claudin5^+^ relative intensity on POD 14. **J** Representative confocal images showing tight junction marker ZO-1 staining on POD 14, scale bar: 20um; **K** Histogram showing quantification of ZO-1^+^ relative intensity on POD 14; One-way ANOVA with Tukey’s multiple comparison test with *n =* 3–4 mice per group. Data presented as mean ± SEM, *P < 0.05, **P < 0.01, ***P < 0.001. CON = control, SEVO = sevoflurane, LAP = laparotomy

We investigated the changes in GLUT1 mRNA levels in the hippocampus at different postoperative time points. While no significant differences were found on POD1, there was a significant decrease in the LAP group compared to the SEVO group on POD 7 and compared to the CON group on POD 14. (Fig. 2A–C, The comparison of GLUT1 mRNA levels on POD14 between young and aged mice was shown in Addtional file 4: B). We then measured GLUT1 expression specifically in isolated cerebral micro-vessel samples from POD 14 and the results showed that microvascular GLUT1 was significantly decreased in the LAP group compared to both the CON and SEVO groups. However, sevoflurane also decreased GLUT1 expression compared to the CON group (Fig. 2D, E). Similarly, immunofluorescent staining of GLUT1 from POD 14 samples also showed a significant reduction in intensity in postsurgical hippocampal tissues but not in those exposed to sevoflurane only (Fig. 2F, G).

We then proceeded to characterize changes in tight junction proteins on POD 14 samples using immunofluorescent staining and showed that hippocampal claudin5 immunoreactivity was decreased in the LAP group, compared to the SEVO and CON groups (Fig. 2H, I), but a reduction of ZO-1 immunoreactivity in the LAP group compared only to the CON group (Fig. 2J–K, ZO-1 negative staining was provided in Additional file 4: A).

These findings revealed that surgical trauma has detrimental effects on GLUT1 and tight junctions in the blood–brain barrier, which may result in dysfunctional glucose intake in the brain and increased blood–brain barrier permeability.

### Targeted metabolomics revealed changes in glucose metabolism in postsurgical aged mice

**Fig. 3:**
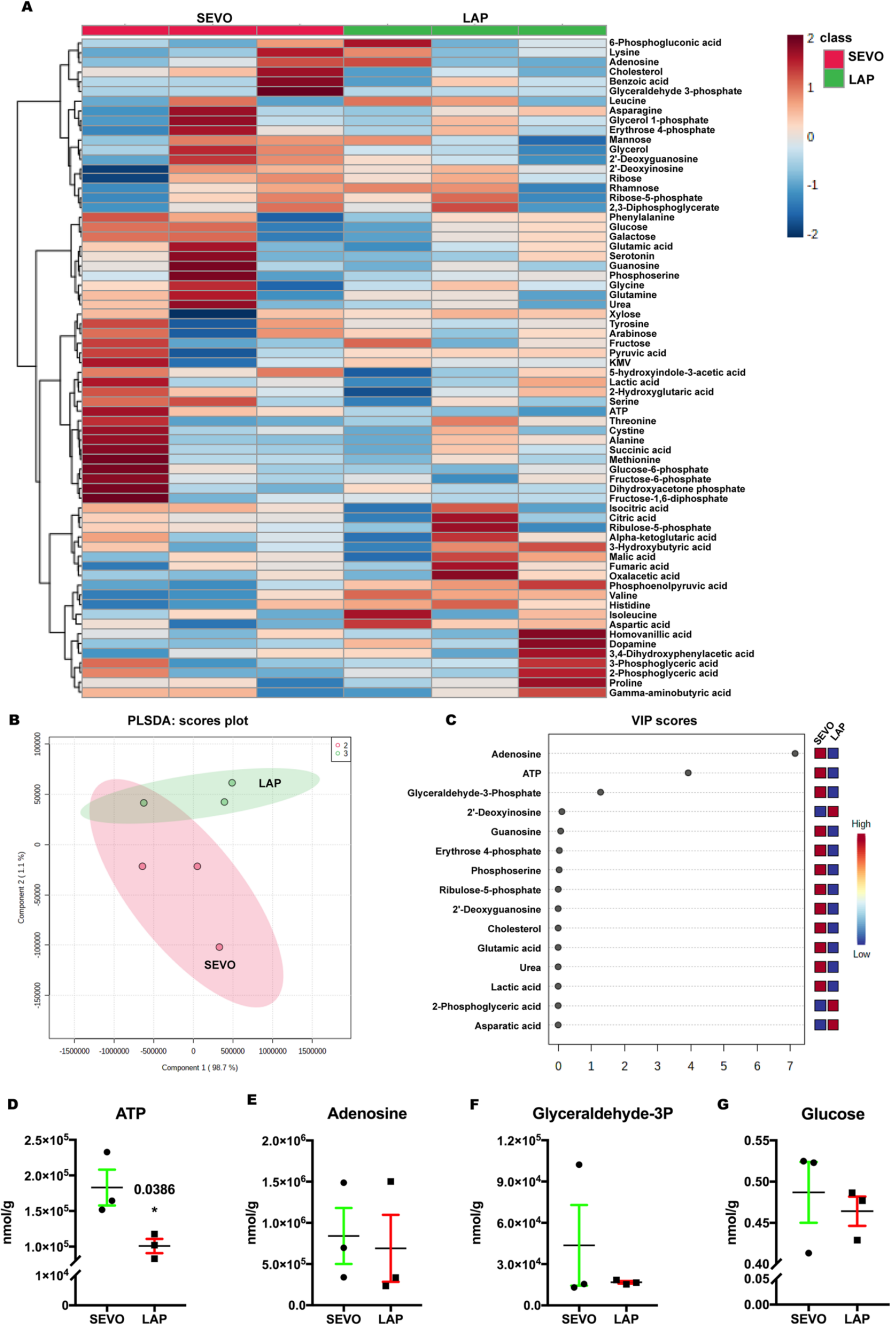
The effects of surgery on the metabolomic profiles in aged mice. **A** Heatmap of metabolites in the postsurgical hippocampus on POD 14. **B** PLSDA scores plot. **C** VIP scores of the PLSDA model. **D**, **E** Histogram showing relative changes in **D** ATP; **E** Adenosine; **F** Glyceraldehyde-3P and **G** glucose; Student’s *t* test with *n =* 3 mice per group. Data presented as mean ± SEM, *P < 0.05. PLSDA: Partial least squares discriminant analysis. VIP scores, variable of importance scores; P, phosphate; SEVO, sevoflurane; LAP, laparotomy

Since a decrease in GLUT1 was found in the postsurgical hippocampus, we further evaluated targeted metabolomics of polar compounds, including glycolytic metabolites, amino acids and neurotransmitters to see whether GLUT1 reduction affected the metabolic activities in the hippocampus. The heatmap displayed the relative changes in metabolites following surgery (Fig. 3A) and discrimination between the postsurgical and sevoflurane only samples emerges when the partial least squares discriminant analysis (PLSDA) was applied (Fig. 3B). Based on the PLSDA results, the variable’s importance scores indicated that adenosine, ATP and glyceraldehyde-3-phosphate are likely the key contributors to this discrimination (Fig. 3C). Further analysis showed a significant decrease of ATP in the LAP group, while no significant differences were found in adenosine, glyceraldehyde-3-phosphate or glucose (Fig. 3D–G). These findings suggested that surgery caused an inadequate energy supply to the hippocampus and this metabolic alteration may be the consequence of GLUT1 downregulation and may contribute to postoperative cognitive dysfunction.

### Conditional overexpression of GLUT1 in cerebral micro-vessels improved tight junction expression and attenuated postoperative cognitive impairment

**Fig. 4:**
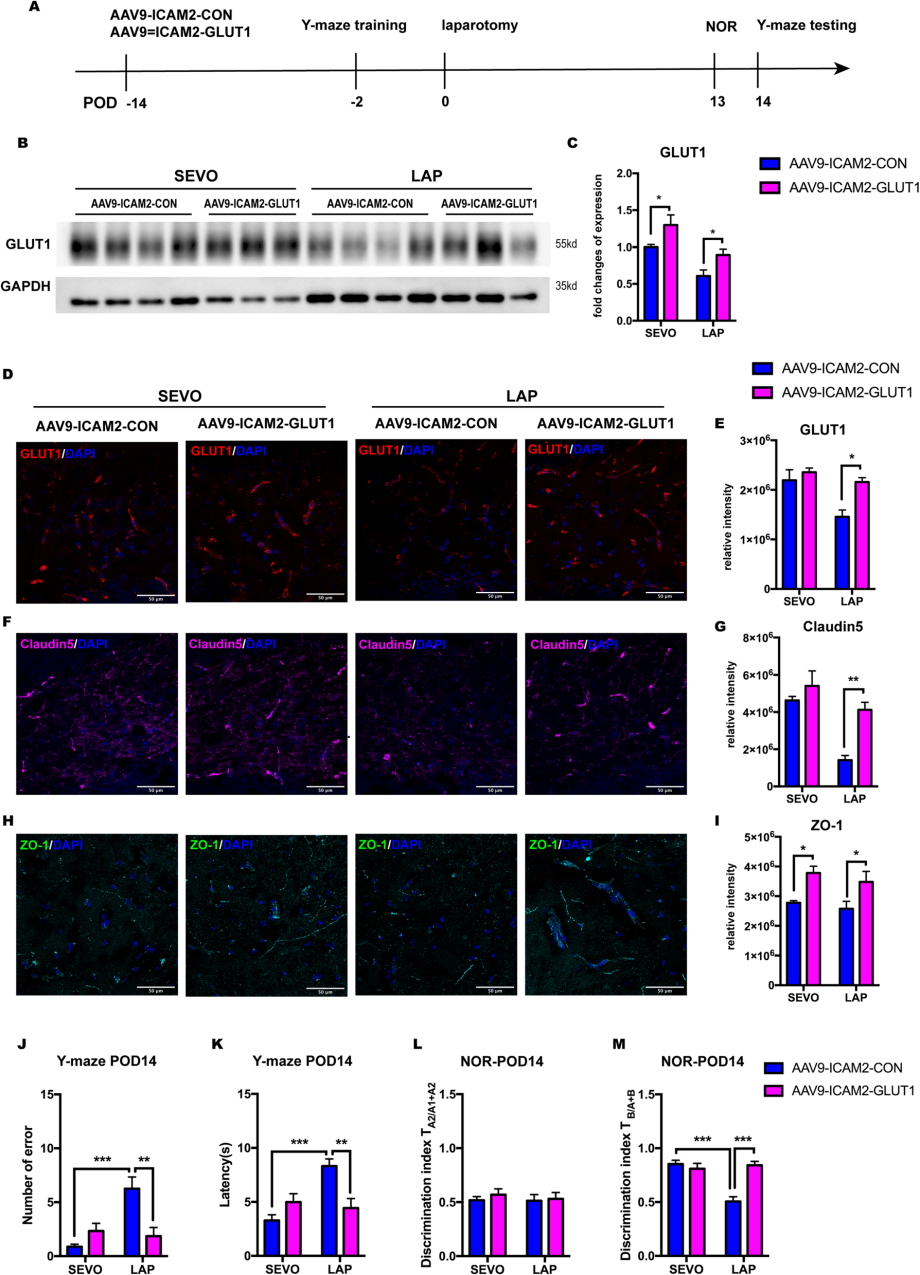
Conditional GLUT1 overexpression in cerebral micro-vessel attenuated postoperative cognitive decline in aged mice. **A** Schema of the experimental design of the GLUT1 intervention. **B** Representative Western blot images of microvascular GLUT1 level in the hippocampus on POD 14. **C** Microvascular GLUT1 expression in the hippocampus, normalized to GAPDH; two-way ANOVA with Tukey’s multiple comparison test with *n =* 6–8 mice per group. **D** Representative confocal images showing GLUT1 staining on POD 14, scale bar: 50um. **E **Histogram showing the quantification of GLUT1^+^ relative intensity. **F** Representative confocal images showing tight junction marker claudin 5 staining on POD 14, scale bar: 50um. **G** Histogram shows quantification of claudin5^+^ relative intensity. **H** Representative confocal images showing tight junction marker ZO-1 staining on POD 14, scale bar: 50um. **I** Histogram shows quantification of ZO-1^+^ relative intensity. Two-way ANOVA with Tukey’s multiple comparison test with *n =* 4 mice per group. **J**, **K** Y-maze test on POD 14 including: the number of errors (**G**) and the latency (**H**), two-way ANOVA with Tukey’s multiple comparison test was applied to the analysis of latency, Kruskal–Wallis test with Dunn’s multiple comparison test was applied to the number of errors with *n =* 6–8 mice per group. **L**, **M** NOR test on POD 14, including: discrimination index of two similar objects (A1 and A2) on POD 13 training (**L**); discrimination index of novel object (B) on POD 14 NOR testing (**M**), two-way ANOVA with Tukey’s multiple comparison test with *n =* 6–7 mice per group. Data is presented as mean ± SEM, *P < 0.05, **P < 0.01, ***P < 0.001. POD, postoperative day; SEVO, sevoflurane; LAP, laparotomy; AAV9-ICAM2-CON,  AAV9 control viral vector with ICAM2 promoter and GFP sequence; AAV9-ICAM2-GLUT1, GLUT1 sequence was inserted into AAV9 control viral vector after ICAM2 promoter and followed by GFP sequence

To further determine the role of microvascular GLUT1 in postoperative cognitive impairment, we conditionally overexpressed endothelial GLUT1 by ICV injection of AAV9-ICAM2-GLUT1 14 days before surgery, and then examined the GLUT1 expression and evaluated the cognitive performance on POD 14 (Fig. 4A). Blood glucose at various time points of the perioperative period was measured. The result showed a drop of 2 mol/L in postoperative blood glucose that did not affect passive glucose transfer into the brain, and that microvascular GLUT1 overexpression did not affect blood glucose in either the SEVO or LAP group, respectively (Additional file 2: A). Furthermore, AAV9-ICAM2-GLUT1 treatment upregulated the microvascular GLUT1 level in the hippocampus of postsurgical mice compared to their counterparts treated with the control vector (Fig. 4B, C). Immunofluorescence staining showed that GLUT1 intensity in the LAP but not SEVO group was increased by AAV9-ICAM2-GLUT1 treatment, which was similar to the GLUT1 protein level (Fig. 4D, E).

Since tight junction proteins were reduced by surgery, we investigated whether GLUT1 overexpression may restore hippocampal tight junction proteins. Immunofluorescent staining showed that GLUT1 overexpression significantly increased hippocampal claudin 5 intensity in the LAP group, while the mild increase in the SEVO group did not reach significance (Fig. 4F, G). GLUT1 overexpression also resulted in a significant increase of hippocampal ZO-1 intensity in both SEVO and LAP groups (Fig. 4H, I). Considering that the reduction of tight junctions correlates with impaired blood–brain barrier integrity, these findings indicates that surgery-induced GLUT1 reduction leads to an impairment in the blood–brain barrier.

Moreover, the behavioral tests showed that microvascular GLUT1 overexpression significantly improved cognitive function in postsurgical mice, with a smaller number of errors and shorter latency in the Y-maze test (Fig. 4J, K) and more interactions with the novel object in NOR test (Fig. 4L, M). In addition, we also examined the effect of GLUT1 overexpression on synaptic protein levels in the hippocampus (Additional file 2: B). The results showed that surgery induced SYN I downregulation but GLUT1 overexpression did not affect this or the expression of other synaptic proteins (Additional file 2: C–F), suggesting GLUT1 overexpression has limited effects on synaptic proteins.

Thus, the increase of cerebral endothelial GLUT1 had protective effects on the blood–brain barrier and cognitive function in postsurgical aged mice, but not synaptic density.

### Enhanced microvascular GLUT1 expression altered brain metabolic profiles in postsurgical aged mice

**Fig. 5:**
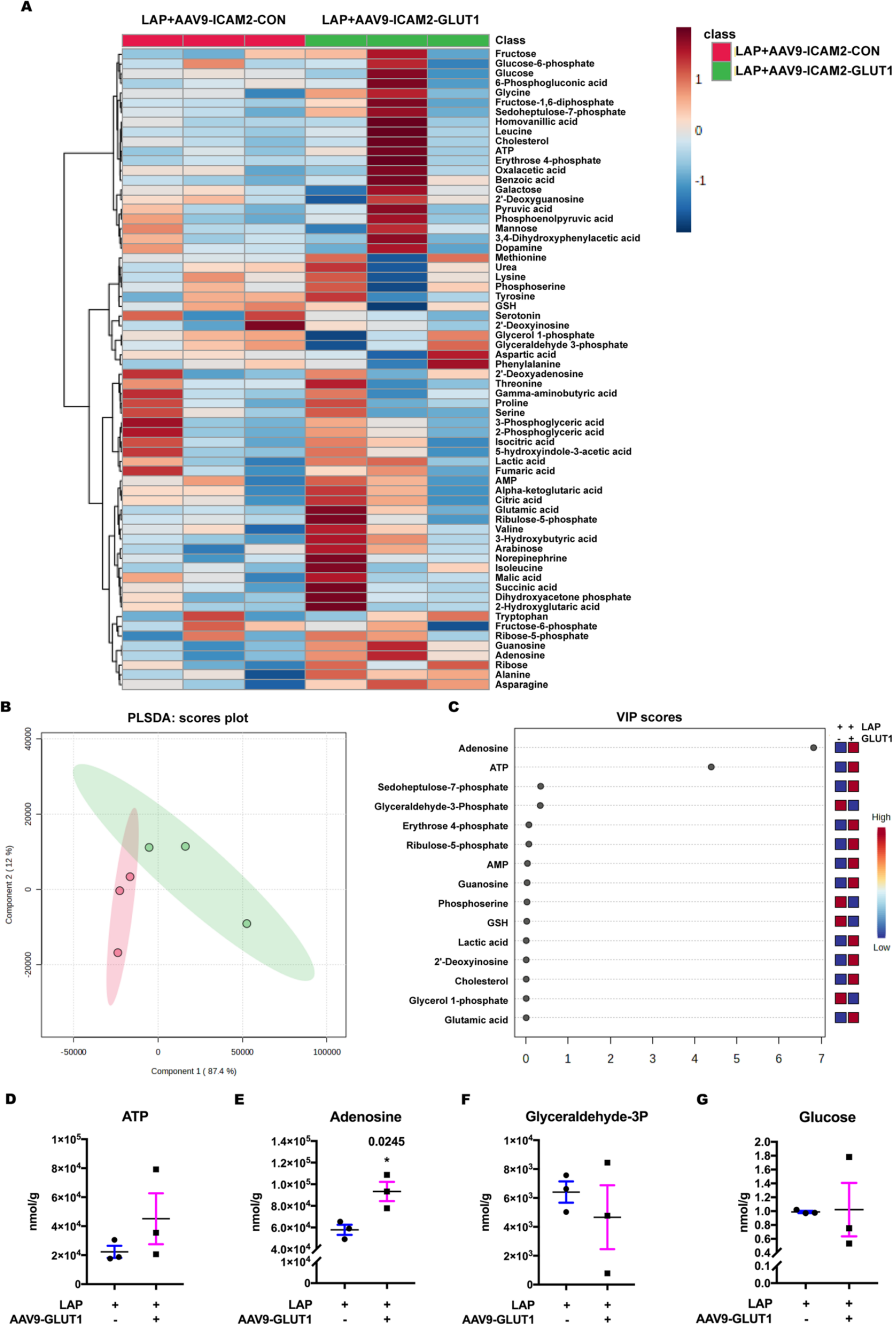
The metabolic profiles after GLUT1 overexpression in postsurgical aged mice. **A** Heatmap of metabolites in the postsurgical hippocampus on POD 14. **B** PLSDA score plot. **C** VIP scores of PLSDA model. **D**-**G** Histogram showing relative changes in ATP (**D**); adenosine (**E**); glyceraldehyde-3P (**F**); and glucose (**G**); Student’s *t* test with *n =* 3 mice per group. Data presented as mean ± SEM, *P < 0.05. PLSDA: Partial least squares discriminant analysis. VIP scores: variable of importance scores. P, phosphate; LAP,  laparotomy; AAV9-ICAM2-CON,  AAV9 control viral vector with ICAM2 promoter and GFP sequence; AAV9-ICAM2-GLUT1 or AAV9-GLUT1,  GLUT1 sequence was inserted into AAV9 control viral vector after ICAM2 promoter and followed by GFP sequence

We further evaluated the effect of microvascular GLUT1 overexpression on targeted metabolomics of polar compounds in the hippocampus of the postsurgical aged mice. The heatmap revealed the different metabolomic profiles in general (Fig. 5A). The PLSDA model revealed the discrimination of brain metabolic profiling between postsurgical aged mice with GLUT1 overexpression and control mice (Fig. 5B). As shown in the VIP scores, ATP and adenosine likely contributed the most to this discrimination, which showed a relative improvement after GLUT1 overexpression (Fig. 5C), and the increase of adenosine, but not ATP, reach statistical significance (Fig. 5D, E). In line with adenosine, guanosine and asparagine were also increased after GLUT1 overexpression (Additional file 2: G, H). Further, neither glyceraldehyde 3-phosphate nor glucose, which was decreased by surgery, showed any significant differences after GLUT1 overexpression (Fig. 5F, G). These findings suggest that GLUT1 overexpression can only partially restore the changes in brain metabolic profiles from surgery in postsurgical aged mice.

### GLUT1 specific inhibitor diminished the protective effects of GLUT1 overexpression in postsurgical aged mice

**Fig. 6:**
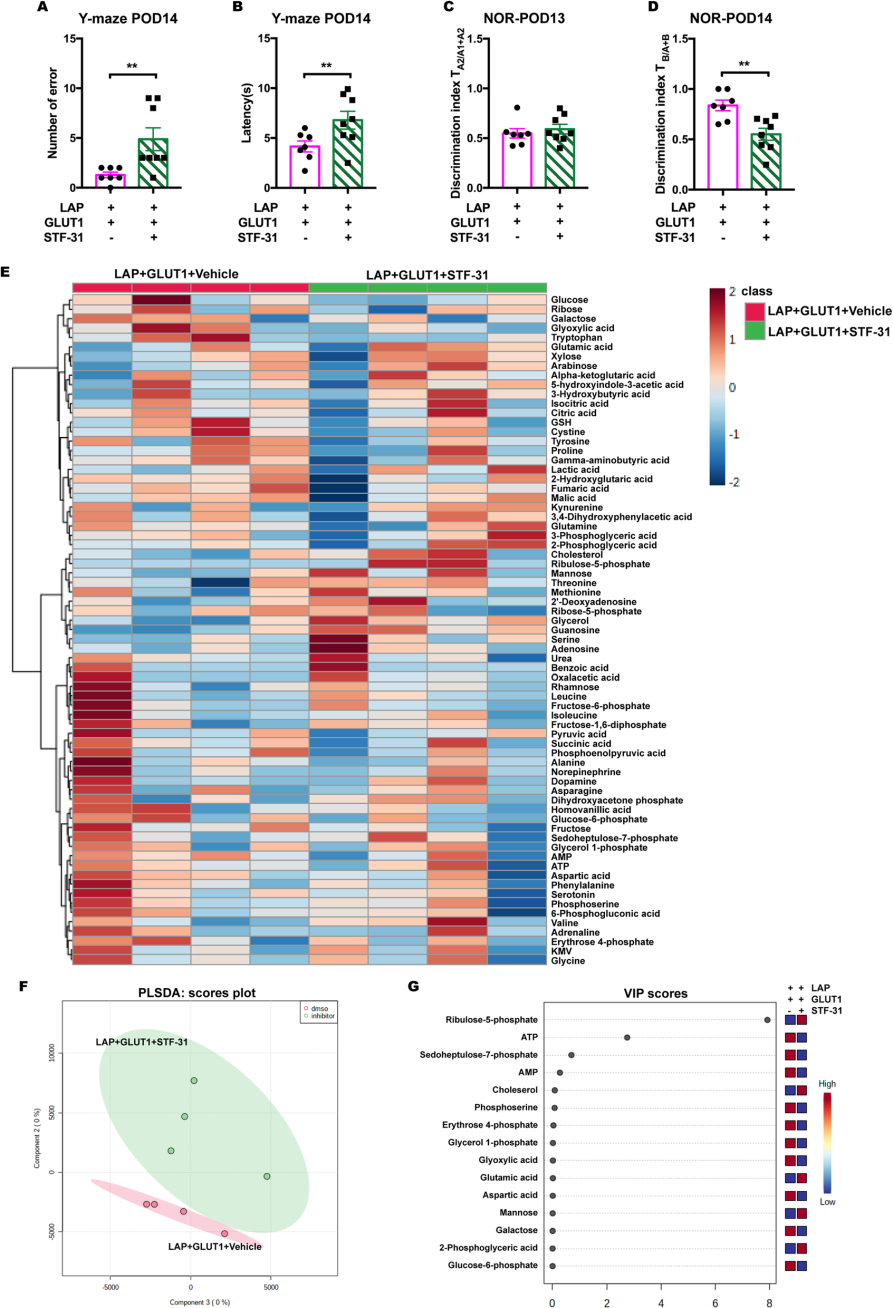
GLUT1 inhibition altered cognitive functions and brain metabolic profiles. **A**, **B** Y-maze test on POD 14, including: the number of errors (**A**) and the latency (**B**), student’s t test applied to the analysis of latency and Mann–Whitney test was applied to the number of errors with *n =* 7–8 mice per group. **C**, **D** NOR test on POD 14, including: discrimination index of two similar objects (A1 and A2) on POD 13 training (**C**); discrimination index of novel object (**B**) on POD 14 NOR testing (**D**), student’s t test with *n =* 7–8 mice per group. **E** Heatmap of metabolites in the postsurgical hippocampus on POD 14. **F**: PLSDA score plot. **G** VIP scores of PLSDA model. Data was presented as mean ± SEM, *P < 0.05, **P < 0.01. LAP, laparotomy; GLUT1,  GLUT1 overexpression by AAV9-ICAM2-GLUT1; STF-31, GLUT1 specific inhibitor; PLSDA, Partial least squares discriminant analysis; VIP scores, variable importance scores

We further investigated the effect of GLUT1 on postoperative cognitive function, by applying the GLUT1 specific inhibitor STF-31 intranasally for 5 days to mice with GLUT1 overexpression and evaluated their behavioral performance and brain metabolism on POD 14. The behavioral results showed that STF-31 significantly abolished the beneficial effects of GLUT1 overexpression, as illustrated by the increased number of errors and latency in Y-maze (Fig. 6A, B) and the decreased interactions in NOR test (Fig. 6C, D). These findings demonstrated the important role of GLUT1 in cognitive performance. Furthermore, the metabolic heatmap displayed the relative changes between mice exposed to STF-31 and their non-exposed counterparts (Fig. 6E). The PLSDA model illustrated that inhibition of GLUT1 changed the metabolic profiling in the hippocampus (Fig. 6F). The VIP scores showed that the increase of riboluse-5-phosphate and the decrease of ATP and sedoheptulose-7-phosphate were the factors most contributory to this discrimination (Fig. 6G). Riboluse-5-phoshpate and sedoheptulose-7-phosphate are the products of pentose phosphate pathway. These results indicated that acute inhibition of GLUT1 resulted in reduced ATP generation, which may also trigger pentose phosphate pathway to compensate ATP supply.

## Discussion

GLUT1, as the major glucose transporter in the blood–brain barrier, is essential for glucose uptake into the brain. Downregulation of GLUT1 in the CNS has been observed in various pathological conditions, which affects glucose metabolism and correlates with cognitive impairment [23, 25]. Our data suggested that GLUT1 is also involved in cognitive impairment induced by surgery. In this study, we demonstrated that surgery-induced downregulation of GLUT1 is linked to impaired cognitive function and diminished hippocampal glucose metabolism, especially ATP generation. Enhanced GLUT1 expression in cerebral micro-vessel alleviated postoperative cognitive decline and altered metabolic profiles in the hippocampus but could not alleviate the decreased ATP levels from surgery. Further application of GLUT1 specific inhibitor abolished this protective effect on cognitive impairment with altered metabolic profiles. Although enhanced GLUT1 expression did not significantly restore hippocampal ATP level, it improved the expression of tight junctions. This study demonstrated that enhanced GLUT1 expression can mitigate postoperative cognitive impairment in aged mice. This may be associated with the improvement in the blood–brain barrier integrity and hippocampal metabolic profiles. Our data provides a possible target to ameliorate postoperative cognitive impairment.

As an important glucose transporter in the brain, GLUT1 reduction can lead to dysregulated glucose metabolism. One of the important functions of glucose metabolism is generating ATP via two pathways: glycolysis and oxidative phosphorylation via the tricarboxylic acid (TCA) cycle. Glycolysis is fast but generates only 2 ATP per glucose molecule, while the net yield of the TCA cycle is 28–30 ATP with a series of oxidative reactions within the mitochondrion. Our findings demonstrated that GLUT1 was downregulated following surgery, accompanied by a significant reduction of ATP levels in the hippocampus. However, enhanced GLUT1 expression did not significantly restore postoperative ATP levels, indicating that the surgery-induced ATP reduction is not entirely dependent on endothelial GLUT1. Our previous study has shown that surgery can induce deficits in mitochondrial density and morphology in aged mice [30]. Thus, post operative mitochondrial dysfunction could be one of the contributors to the depressed ATP level. Less-glycosylated GLUT1 with the molecular mass of 45 kDa is expressed on the end-feet of astrocytes that surrounded by capillaries [21, 24]. Astrocytic GLUT1 can take up blood glucose from capillaries, convert them into ATP or lactate and supply them to neurons [21, 34]. The generation of ATP or lactate in astrocytes are important for neuronal activities and critical for memory formation [34, 35]. Astrocytic GLUT1 may also be suppressed by surgery, which can further affect total ATP levels in the brain. However, our study did not enhance astrocytic GLUT1. This may account for the lack of significant improvement in ATP level by endothelial GLUT1 overexpression.

Although enhanced microvascular GLUT1 did not restore ATP levels, it resulted in altered metabolic profiles with some neuroactive metabolites being improved, which may have contributed to mitigating postoperative cognitive impairment. Adenosine, a metabolic intermediate, can be released by neurons or astrocytes and act as a neuromodulator. Our results showed that statistically significant increase in adenosine in the hippocampus following GLUT1 overexpression. Adenosine is involved in sleep–wake regulation, coordinating with other neurotransmitters and neuromodulators [36], and recent studies also showed that adenosine and its receptors affect cognitive function. Local adenosine-augmented treatments significantly improved memory deficits in schizophrenia like phenotypic mice [37]. Furthermore, stimulation of A3 adenosine receptor by specific agonist improved neurocognitive function after traumatic brain injury [38]. The same agonist also protects against cisplatin-induced neurotoxicity and improves cognitive impairment in mice [39]. Interestingly, antagonising the A2A adenosine receptor also prevents cisplatin-induced impairments in neurogenesis and cognitive function [40]. These results indicate that adenosine has complicated and multiple effects on brain functions, which depended on various subtypes of adenosine receptors. Guanosine, another endogenous nucleoside, also had significant improvement after GLUT1 overexpression. Guanosine administration mitigates sepsis-induced brain damage and cognitive impairment in rats by decreasing oxidative stress in the brain [41]. Another study also reported protective effects of guanosine treatment in traumatic brain injury by promoting mitochondrial function [42]. Interestingly, exogenous guanosine interacts with adenosine receptors and promote SUMOylation in astrocytes or neurons, which is a post-translational modification implicated in neurodegeneration [43]. In line with these observations in other neurotoxic models, the improvement of postoperative cognitive function following GLUT1 overexpression seen here maybe the result of augmenting adenosine or guanosine levels, but this requires further confirmatory studies.

Apart from GLUT1, tight junction proteins such as claudin 5 and ZO-1 were also affected by surgery. Interestingly, GLUT1 overexpression augmented the expression of claudin 5 in postsurgical mice only and ZO-1 in mice exposed to sevoflurane and postoperatively. These changes in tight junction proteins are in keeping with GLUT1 changes in this study and are consistent with observations from GLUT1 knock-out in AD transgenic mice, where decreasing tight junction proteins and early breakdown of the blood–brain barrier are also seen [25].

GLUT1 expression or activity is independent of insulin signaling, while hypoglycemia can induce GLUT1 upregulation with no change in glucose uptake [44]. Our results showed that surgery induced mild but sustained hypoglycemia during the postoperative period (Additional file 1: Fig. S1, Fig. 4B), but GLUT1 was surprisingly lower. This persistent GLUT1 reduction in postsurgical aged mice may have also led to dysregulated glucose metabolism within endothelial cells. A recent study reported that acute and severe hypoglycemia caused blood–brain barrier dysfunction with impaired tight junctions, which further contributes to cognitive decline [45]. Another study suggested that this hypoglycemia-related impairment could be due to increased mitochondrial oxidative stress [46]. These results indicate that the change of tight junctions is sensitive to blood glucose level and correlate with cognitive changes. Furthermore, some metabolites can mediate claudin 5 expression. For example, fluid containing alanyl-glutamine increased claudin 5 expression, and also restored its distribution in human endothelial cells [47]. Another lipid component, trans-10-hydroxy-2-decenoic acid (10-HA), indirectly protected claudin 5 and other tight junction expression by alleviating inflammatory responses and decreasing matrix metalloproteinases (MMPs) [48]. Considering that tight junctions are vulnerable to hypoglycemia or to different levels of different metabolites, enhanced GLUT1 expression may possibly preserve endothelial glucose metabolism and consequent improvement of tight junctions. However, further studies are required to confirm this hypothesis.

## Conclusions

Taken together, our results demonstrated that endothelial GLUT1 is downregulated in the postoperative period, which correlated to impaired cognitive function and dysregulated hippocampal glucose metabolism, especially ATP generation. Enhanced GLUT1 expression alleviated cognitive dysfunction with increased expression of tight junction proteins and altered metabolic profiles. GLUT1 specific inhibitor abolished this protective effect of GLUT1 overexpression on cognitive function with alter metabolic profiles. These findings demonstrated that GLUT1 downregulation play an important role in postoperative cognitive impairment. This gives further supports to develop a therapeutic strategy targeting GLUT1 to improve metabolic profile, blood–brain barrier integrity and cognitive function.

### Supplementary Information

**Additional file 1. A** Changes in blood glucose during the postoperative period. Two-way repeated ANOVA with Tukey’s multiple comparison test with *n =* 8 mice per group. **B**, **C** Open field test on POD 4, total distance (cm) during 10 min observation (**B**), the duration time in the central area (**C**). **D**, **E** Y-maze test on POD 7 including: the number of errors (**D**); the latency (**E**). One-way ANOVA with Tukey’s multiple comparison test was applied to the analysis of latency, Kruskal-Wallis test with Dunn’s multiple comparisons test was applied to the number of errors with *n =* 6-8 mice per group on POD7. **F**, **G** NOR test on POD 7, including: discrimination index of two similar objects (A1 and A2) on POD 6 training (**F**); discrimination index of novel object (B) on POD 7 testing (**G**). One-way ANOVA with Tukey’s multiple comparison test with *n =* 6-8 mice per group. **H** Histogram showing total interaction of axon with concentric circles, **I:** Histogram showing total branch length, **J** Histogram showing total branch number, One-way ANOVA with Tukey’s multiple comparison test, 6 neurons per mice were analysed, *n =* 4 mice per group. Data was presented as mean ± SEM, *P < 0.05, **P < 0.01, ***P < 0.001. Data is presented as mean ± SEM, *P < 0.05, **P < 0.01, ***P < 0.001. POD, post-operative day; Y-maze test,  forced alternation Y-maze test; NOR test,  novel object recognition test; CON, control; SEVO, sevoflurane; LAP,  laparotomy.

**Additional file 2.** **A** The changes in blood glucose at different timepoints during the postoperative period. Two-way repeated ANOVA with Tukey’s multiple comparison test with *n =* 12 mice per group*.* **B** Representative WB images of pre- and post-synaptic markers from the hippocampal synaptosome on POD 14. **C–F** statistical analysis of pre- and post-synaptic protein levels including: PSD95 (**C**), SYN I (**D**), SYP (**E**), and SYB (**F**), normalized to β-tubulin, two-way ANOVA with Tukey’s multiple comparison test with *n =* 4 mice per group. Data was presented as mean ± SEM, *P < 0.05, **P < 0.01, ***P < 0.001. **G**, **H** Histogram showing relative changes in guanosine (**G**) and asparagine (**H**), Student’s t test with *n =* 3 mice per group. Data was presented as mean ± SEM. TUJ, β-tubulin; SEVO, sevoflurane; LAP,  laparotomy; AAV9-ICAM2-CON,  AAV9 control viral vector with ICAM2 promoter and GFP sequence; AAV9-ICAM2-GLUT1 or AAV9-GLUT1,  GLUT1 sequence was inserted into AAV9 control viral vector after ICAM2 promoter and followed by GFP sequence.

**Additional file 3.** All uncropped blot images.

**Additional file 4. A** The negative staining images of ZO-1 (Goat anti Rabbit IgG (H + L) Cross-Adsorbed Secondary Antibody Alexa Fluor^®^ 488 conjugate was used). **B** The comparison in fold changes of GLUT1 mRNA levels between young and aged mice, surgery induced a significant reduction of GLUT1 in aged mice but not in young mice. The postoperative GLUT1 expression was decreased in aged mice compare to young counterparts, while young and aged mice in the CON group had similar levels of GLUT1 expression.

## Data Availability

Experimental data reported in this paper will be shared by the corresponding authors upon request.

## Abbreviations

AAV: Adeno-associated virus
AD: Alzheimer’s disease
ATP: Adenosine triphosphate
BBB: Blood-brain barrier
CNS: Central nervous system
GLUT1: Glucose transporter 1
GFP: Green fluorescent protein
ICAM: Intercellular adhesion molecule
MMPs: Matrix metalloproteinases
NOR: Novel object recognition
PLSDA: Partial least squares discriminant analysis
OFT: Open field test
PNDs: Perioperative neurocognitive disorders
POD: Post-operative day
TCA: Tricarboxylic acid
VIP: Variable importance
