# Supplementary figures and images for: Downregulation of the glucose transporter GLUT 1 in the cerebral microvasculature contributes to postoperative neurocognitive disorders in aged mice
Source: J Neuroinflammation. 2023 Oct 19;20:237. doi: 10.1186/s12974-023-02905-8 (PMC10588063; doi:10.1186/s12974-023-02905-8)

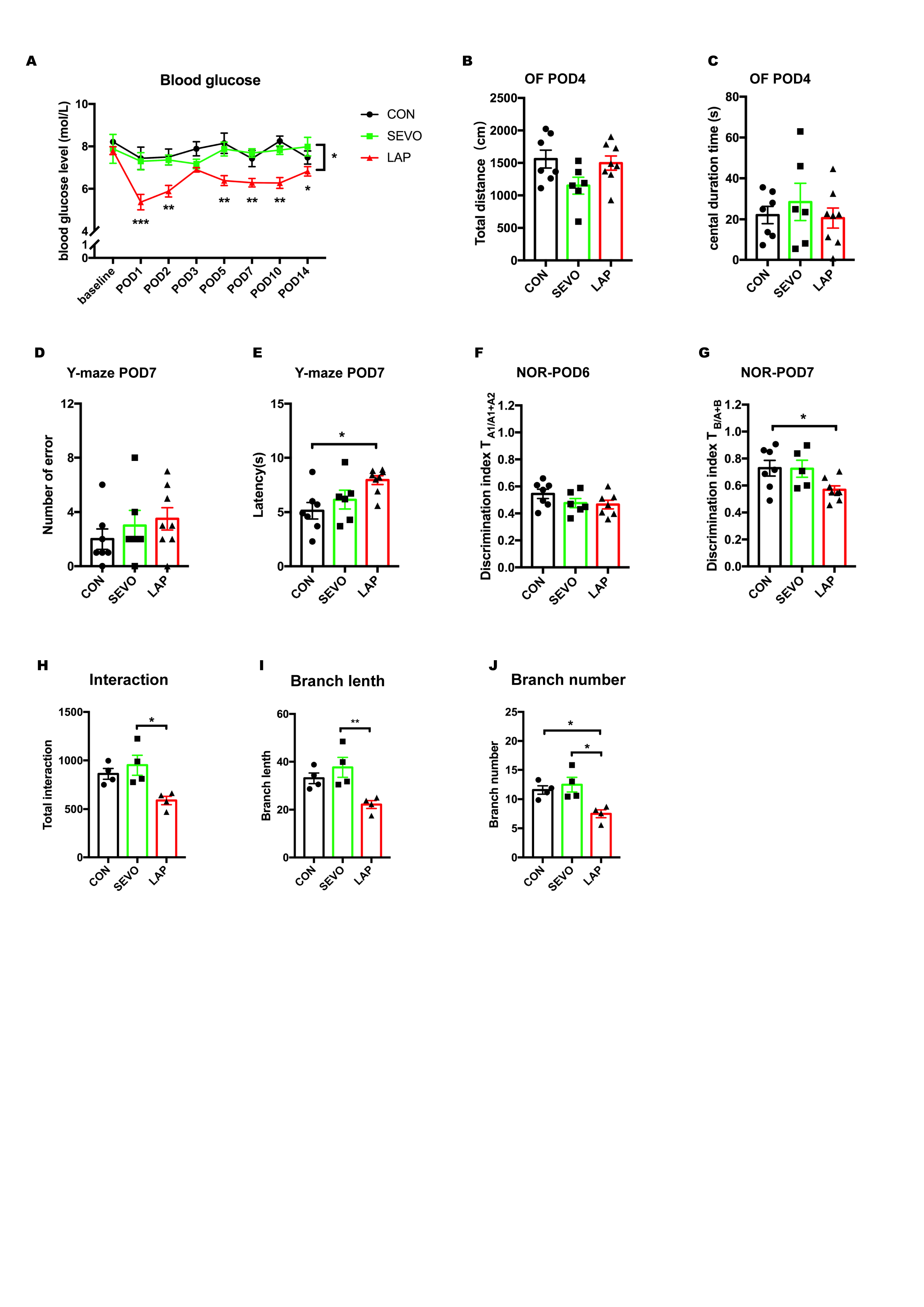

Supplement: Supplementary file 1 — Additional file 1. A Changes in blood glucose during the postoperative period. Two-way repeated ANOVA with Tukey’s multiple comparison test with n = 8 mice per group. B, C Open field test on POD 4, total distance (cm) during 10 min observation (B), the duration time in the central area (C). D, E Y-maze test on POD 7 including: the number of errors (D); the latency (E). One-way ANOVA with Tukey’s multiple comparison test was applied to the analysis of latency, Kruskal-Wallis test with Dunn’s multiple comparisons test was applied to the number of errors with n = 6-8 mice per group on POD7. F, G NOR test on POD 7, including: discrimination index of two similar objects (A1 and A2) on POD 6 training (F); discrimination index of novel object (B) on POD 7 testing (G). One-way ANOVA with Tukey’s multiple comparison test with n = 6-8 mice per group. H Histogram showing total interaction of axon with concentric circles, I: Histogram showing total branch length, J Histogram showing total branch number, One-way ANOVA with Tukey’s multiple comparison test, 6 neurons per mice were analysed, n = 4 mice per group. Data was presented as mean ± SEM, *P < 0.05, **P < 0.01, ***P < 0.001. Data is presented as mean ± SEM, *P < 0.05, **P < 0.01, ***P < 0.001. POD, post-operative day; Y-maze test, forced alternation Y-maze test; NOR test, novel object recognition test; CON, control; SEVO, sevoflurane; LAP, laparotomy. [file 12974_2023_2905_MOESM1_ESM.tif]

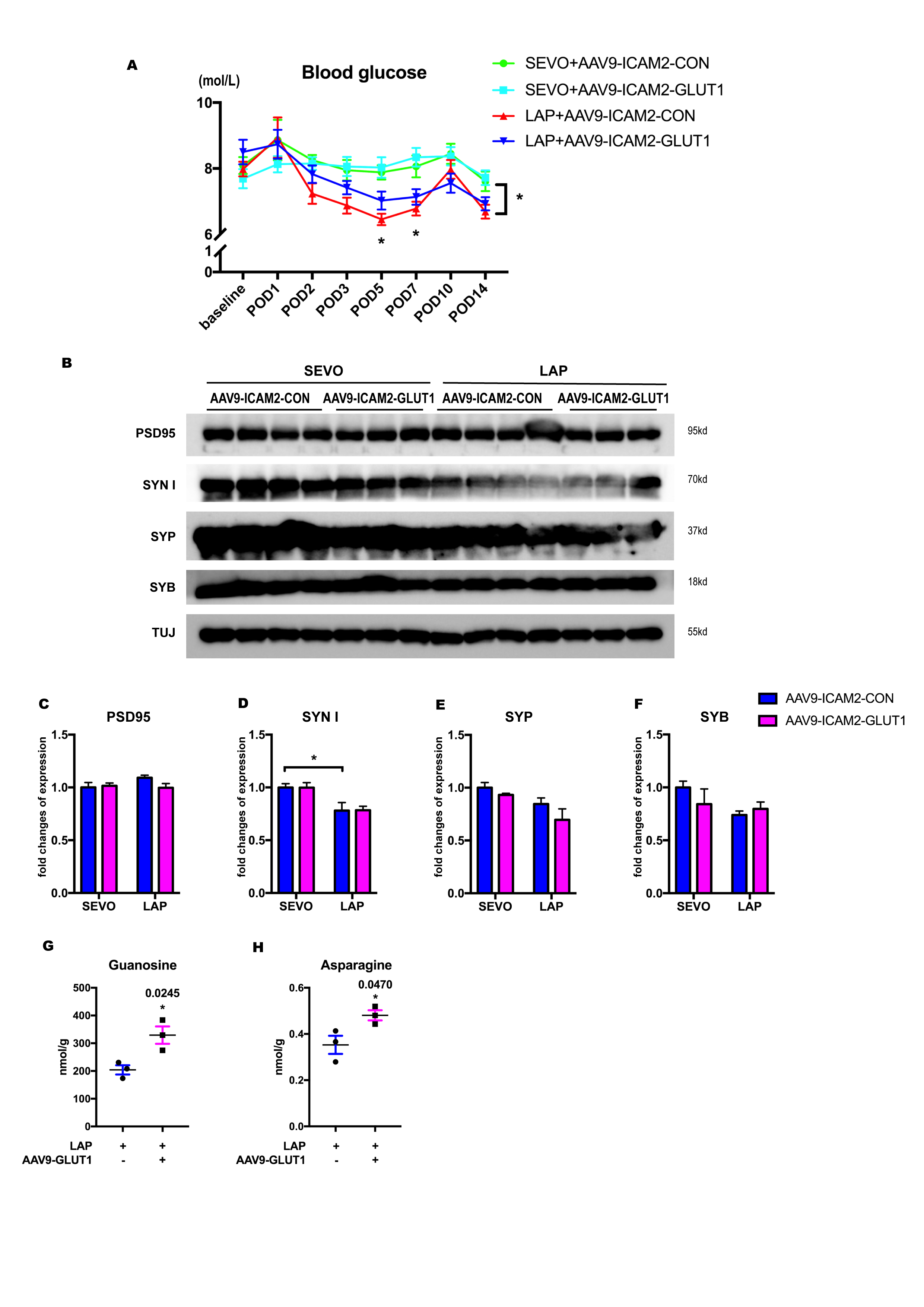

Supplement: Supplementary file 2 — Additional file 2. A The changes in blood glucose at different timepoints during the postoperative period. Two-way repeated ANOVA with Tukey’s multiple comparison test with n = 12 mice per group. B Representative WB images of pre- and post-synaptic markers from the hippocampal synaptosome on POD 14. C–F statistical analysis of pre- and post-synaptic protein levels including: PSD95 (C), SYN I (D), SYP (E), and SYB (F), normalized to β-tubulin, two-way ANOVA with Tukey’s multiple comparison test with n = 4 mice per group. Data was presented as mean ± SEM, *P < 0.05, **P < 0.01, ***P < 0.001. G, H Histogram showing relative changes in guanosine (G) and asparagine (H), Student’s t test with n = 3 mice per group. Data was presented as mean ± SEM. TUJ, β-tubulin; SEVO, sevoflurane; LAP, laparotomy; AAV9-ICAM2-CON, AAV9 control viral vector with ICAM2 promoter and GFP sequence; AAV9-ICAM2-GLUT1 or AAV9-GLUT1, GLUT1 sequence was inserted into AAV9 control viral vector after ICAM2 promoter and followed by GFP sequence. [file 12974_2023_2905_MOESM2_ESM.tif]

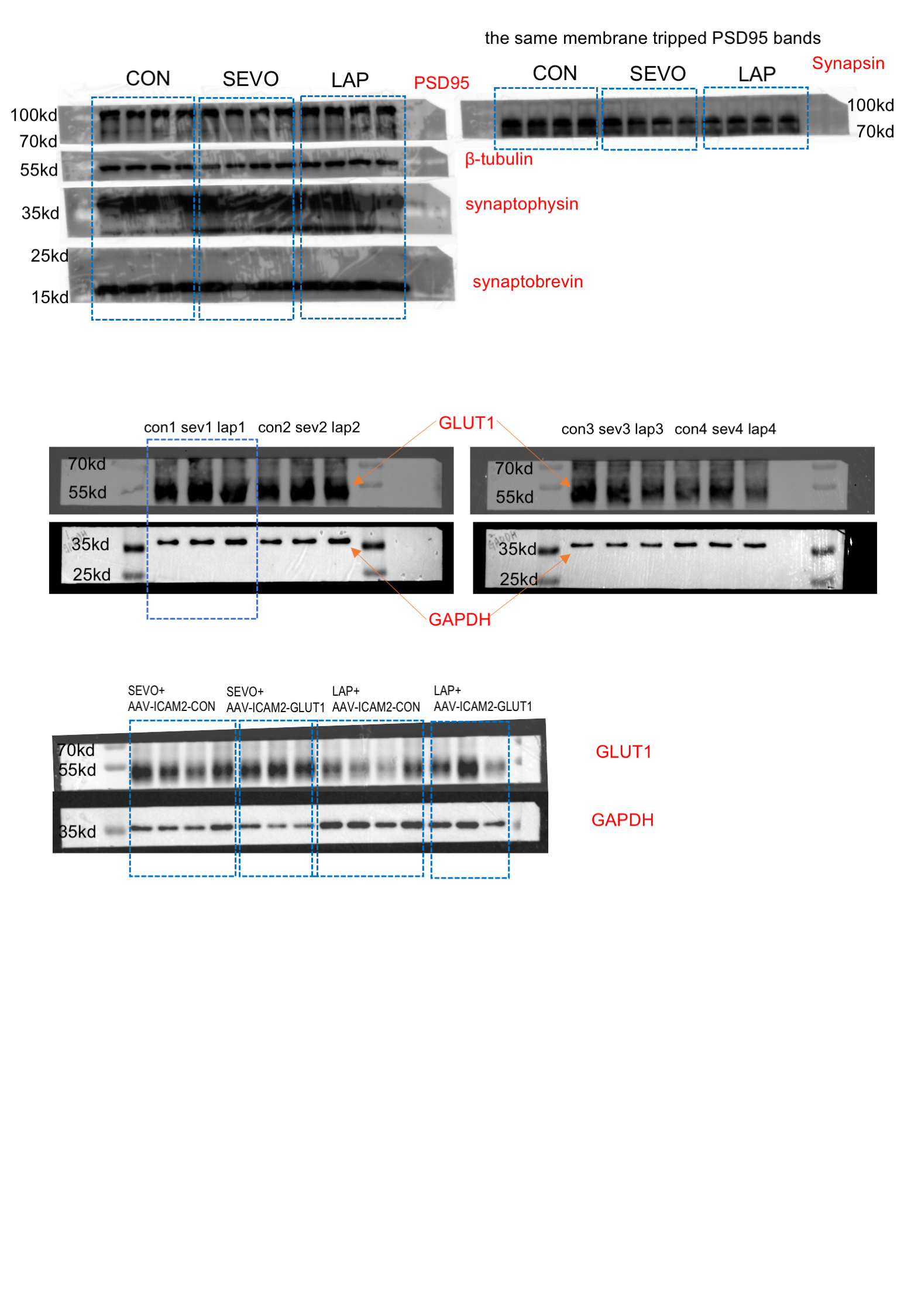

Supplement: Supplementary file 3 — Additional file 3. All uncropped blot images. [file 12974_2023_2905_MOESM3_ESM.tiff]

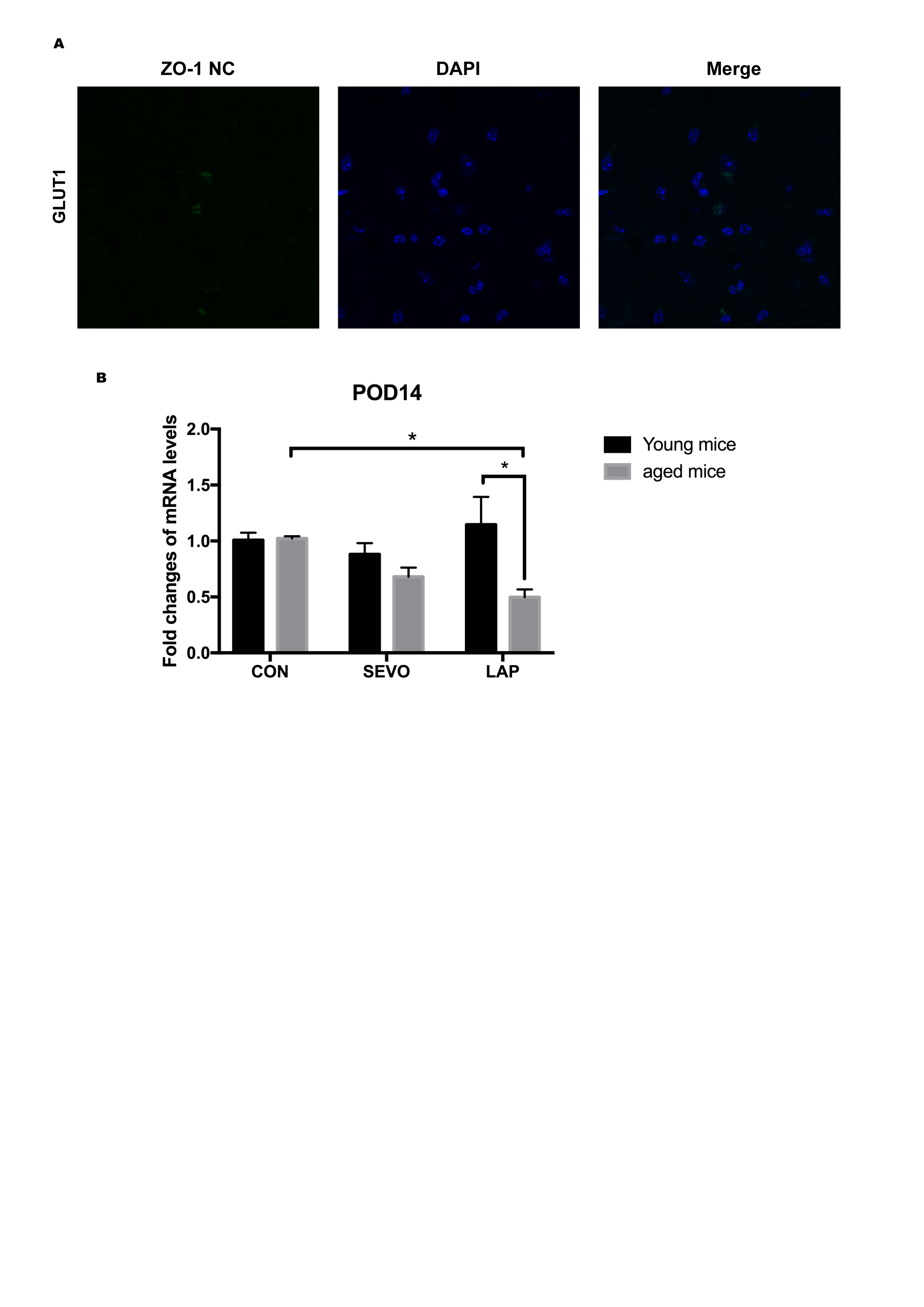

Supplement: Supplementary file 4 — Additional file 4. A The negative staining images of ZO-1 (Goat anti Rabbit IgG (H + L) Cross-Adsorbed Secondary Antibody Alexa Fluor® 488 conjugate was used). B The comparison in fold changes of GLUT1 mRNA levels between young and aged mice, surgery induced a significant reduction of GLUT1 in aged mice but not in young mice. The postoperative GLUT1 expression was decreased in aged mice compare to young counterparts, while young and aged mice in the CON group had similar levels of GLUT1 expression. [file 12974_2023_2905_MOESM4_ESM.tif]
